# A closed loop fully automated wireless vagus nerve stimulation system

**DOI:** 10.1038/s41598-025-11159-8

**Published:** 2025-07-30

**Authors:** Roshan Pathitharayil Mathews, Iman Habibagahi, Hamid Jafari Sharemi, Ronald Challita, Steven Cha, Aydin Babakhani

**Affiliations:** 1 Electrical and Computer Engineering, University of California Los Angeles, Los Angeles, CA USA; 2 UCLA Cardiac Arrhythmia Center and Neurocardiology Research Program of Excellence, David Geffen School of Medicine, University of California Los Angeles, Los Angeles, CA USA

**Keywords:** Electrical and electronic engineering, Biomedical engineering, Peripheral nervous system

## Abstract

Vagus nerve stimulation (VNS) has been explored as a treatment for a range of conditions, including epilepsy, cardiovascular disorders, drug-resistant depression, chronic pain, and obesity. Conventionally, VNS is administered using an open-loop approach, in which trained personnel adjust stimulation parameters. Medical supervision is necessary to minimize adverse effects, such as severe bradycardia, which can significantly interfere with daily activities. This requirement limits the feasibility of VNS in unsupervised settings and highlights the need for an automated control system. To address this limitation, we introduce the fully automated wireless VNS (FAW-VNS) system, which dynamically adjusts stimulation parameters to maintain steady-state operation while minimizing bradycardia. The FAW-VNS system operates in real-time and consists of a biocompatible, miniaturized, wirelessly powered implant equipped with cuff electrodes; a handheld device for power delivery and stimulation protocol communication; a sensing patch to collect and transmit heart rate (HR) data; and a central control unit (CCU) that updates stimulation protocols based on the acquired physiological signals. In-vivo studies were conducted on four anesthetized pigs to validate the system’s ability to reach a steady-state response, achieving a controlled HR reduction within 2–4% of baseline during stimulation. This work lays the foundation for developing closed-loop, wireless implants for point-of-care applications.

## Introduction

The vagus nerve is a principal component of the parasympathetic autonomic nervous system. VNS has been investigated as a therapeutic intervention for various conditions, including drug-resistant epilepsy, heart failure, ventricular and atrial arrhythmias, myocardial infarction (MI), and stroke. Initially, VNS was hypothesized to mitigate excessive cerebral blood flow associated with epilepsy and was later demonstrated to modulate brain electrical activity, thereby terminating seizures^1^. The first human clinical trials in 1994 established the anti-epileptic potential of VNS, leading to its FDA approval for the treatment of refractory epilepsy in 1997^1^. In the context of heart failure, it is postulated that an imbalance between the sympathetic and parasympathetic nervous systems, characterized by sympathetic overactivation, results in afferent sympathetic signaling that reflexively inhibits cardiac vagal efferent activity, thereby exacerbating heart failure. Low-level VNS has been shown to positively impact sympathetic drive, lowering pro-inflammatory cytokines, nitrous oxide elaboration, and myocardial expression of gap junction proteins, all of which may contribute to improved cardiac function^1^. Similarly, in atrial fibrillation (AF), low-level VNS has been shown to stimulate the cholinergic pathway and inhibit the intrinsic cardiac nervous system, thereby reducing AF inducibility^1,2^. In stroke, where neuroinflammatory injury is a key contributor to acute neuronal damage, VNS has been implicated in the regulation of both peripheral and central inflammation, thus offering therapeutic potential for stroke management. Although VNS is associated with clinically significant improvements, several adverse effects have been reported. The most prominent among these is bradycardia, with possible asystole^1^. Other side effects of VNS include laryngeal muscle activation leading to hoarseness, reduced vocal cord mobility^3^, and alterations in respiratory rate^4^. In this work, we regulate VNS to minimize the negative side effects, primarily bradycardia, to ensure its safe application in daily life.

The efficacy of VNS in integrated cardiac control is well-documented in the literature^5–7^. The term “neural fulcrum” refers to an operating point at which a null change in HR response is generated during the active phase of the VNS. Altering the stimulation parameters from the neural fulcrum results in either bradycardia (with increased stimulation intensity) or tachycardia^5,6^. The neural fulcrum can be obtained from either the left VNS (LVNS) or right VNS (RVNS), and the HR response remains consistent across both awake and anesthetized states. In ref.^5^, the authors highlight the need for an implantable pulse generator (IPG) with HR sensing capabilities for automatic neural fulcrum detection and closed-loop control. Closed-loop operation is crucial for point-of-care applications, as it addresses limitations of traditional open-loop systems by enabling adaptive, patient-specific stimulation parameters^8–10^. Such systems enable safer and more efficient stimulation by continuously monitoring and responding to patients’ well-being. In recent years, significant progress has been made in developing real-time closed-loop VNS systems^11–17^. Prior art has explored the use of closed-loop VNS in applications such as AF^13,14,18^, epilepsy^17^, and HR modulation^11–16,19,20^.

Several control strategies have been proposed to modulate HR using VNS, including human-in-the-loop^18^, cumulative sum (CUSUM)^13^, bang-bang (on-off)^16^, proportional-integral (PI)^19^, state transition (ST)^15^, and Fuzzy Logic Control (FLC)^20^. The work in ref.^18^ uses a human in the loop to control the VNS based on the onset of AF. An ECG sensor records and transmits signals to a central control unit (CCU), which detects the onset of AF. Upon detection, an alarm prompts the user to press a self-powered nanogenerator that powers the vagus nerve stimulator. While this represents a form of closed-loop VNS, it lacks full automation and relies on the patient’s immediate action while experiencing AF to activate the VNS. The paper in ref.^13^ uses a CUSUM controller to adjust the stimulation frequency based on the cumulative sum of prior frequencies and deviations of HR from a target value. Although this controller maintains a selected target HR during AF, the demonstrated approach is limited by its ability to tune only a single parameter, which can limit its applicability. The study in ref.^16^ focuses on controlling the HR using a bang-bang VNS control. The bang-bang stimulator produces large overshoots in stimulation response and coarse adjustments in HR. Additionally, the prolonged active phase required for HR control increases stress on the animal during the experiment. In ref.^19^, a PI controller is used to demonstrate a closed-loop VNS. The PI controller was initially designed using an in-silico model of the cardiac effects of VNS and validated on an ex-vivo isolated rabbit heart. However, the proportional-integral-derivative (PID) family of controllers is inherently sensitive to inter-animal variability and model inaccuracies and can become unstable when the physiological response to VNS changes over time^20^. This can potentially cause ringing or large overshoots in stimulation parameters.

The work in ref.^15^ implements a novel ST controller where the outputs are based on a pre-trained mapping of the different states (VNS stimulation parameters). Using this learned mapping, the algorithm predicts the subsequent state based on the current state and the corresponding HR response. The simplicity of the model makes it well-suited for low-power implantable devices. However, the drawbacks of such ST models are that they need 1) a significant number of states to achieve accurate control and 2) a good calibration/training phase. As model dynamics evolve, training and recalibration become challenging, particularly for a large number of states. In contrast to fixed-state models, most biomedical applications rely on empirical data and clinical observation to define a working state. Consequently, fuzzy logic has gained traction for such control system requirements. The work in ref.^20^ develops an FLC-based control system to regulate the HR to a set-point during VNS by alternating between kilohertz frequency alternating current (KHFAC) nerve block and nominal VNS paradigm depending on the HR response. The fuzzy logic was designed using empirical approaches upon observing the animal’s response to both the KHFAC waveform and stimulation parameters. Although FLCs provide a superior alternative over PID/STs, the definition of the fuzzy states based on human expertise makes tuning difficult and increases computational complexity during model deployment. Moreover, all the aforementioned automated VNS studies focus on achieving a target HR value through adaptive VNS control using conventional VNS hardware, which is bulky and tethered.

Over the past decade, implantable electronics have played a pivotal role in transitioning from costly and time-consuming clinical treatments to automated point-of-care solutions. IPGs are widely used in clinical practice, with common examples including pacemakers for cardiac control^8,21,22^, vagus nerve stimulators for epilepsy and cardiac therapy^23–26^, and deep brain stimulation (DBS)^27,28^. Miniaturized, wireless, and batteryless IPGs offer crucial advantages such as preserving the natural movement and behaviors of animals post-surgery, reducing the risk of infections, and enhancing biocompatibility^10,29,30^. The removal of bulky batteries from implants necessitates alternative power sources. Several strategies have been proposed to harvest energy from the animal’s natural movements^25,30,31^. For instance, ref.^25^ demonstrates successful weight control using VNS, with power harvested from a triboelectric nanogenerator. While this approach simplifies system design with millimeter-sized implants, the harvested power (approximately 100 nW) is significantly lower than the required power (approximately 50 $\upmu$W) for electrical stimulation in epilepsy and cardiac pacing^30,32,33^. Furthermore, the dependence on mechanical movement for power harvesting makes this method less desirable for critical applications such as heart pacing and epilepsy^30^.

Wireless power transfer (WPT) is essential to achieve the required power and robustness in a batteryless system^10,30^. Despite its advantages, WPT also presents challenges, such as compliance with radiation safety limits and the physical size of the implant^34,35^. Recent advancements in WPT methods, including ultrasound (US)^29,36^, magnetoelectric (ME)^37,38^, and hybrid magnetic-ultrasonic approaches^39^, have improved WPT efficiency while retaining comparable dimensions to traditional inductive systems. Nevertheless, each of these emerging techniques has inherent limitations. The US method often requires direct contact and the application of gel on the skin, limiting its practicality in various applications^29,40^. Moreover, US experiences significant attenuation when traversing different media interfaces, such as air to skin, muscle, or bone^21^. The ME technique relies on a composite of magnetostrictive and piezoelectric layers, which demands precise and costly fabrication processes^37,39^. Additionally, both US and ME methods are further constrained by their operation in sub-MHz frequency bands, resulting in limited data communication bandwidth^37,39^. Despite recent advances in WPT techniques that offer high efficiency and implant miniaturization, non-radiative inductive coupling (NRIC) remains the gold standard for powering batteryless bioelectronic systems, primarily due to its reproducibility and simplicity, making it highly suitable for large-scale manufacturing^30,41^. In this work, we employ a batteryless VNS implant based on the resonant NRIC technique^42,43^.

In this work, following extensive open-loop experiments, we have developed a customized state-based algorithm to enable rapid and multiparametric control of VNS. The primary stimulation parameters of interest are frequency, pulse width (PW), and pulse duty cycle, which are used to reach the neural fulcrum. This study presents the first realization of batteryless VNS technology operating in a closed-loop configuration. We introduce a fully automated and wireless VNS system (FAW-VNS) designed to perform real-time closed-loop stimulation. The FAW-VNS system comprises four key components: (I) a biocompatible, miniaturized, wirelessly powered implant with cuff electrodes; (II) a handheld device for powering the implant and relaying the stimulation protocol; (III) a wearable sensing patch with a microcontroller to obtain and transmit electrocardiogram (ECG) data; and (IV) a central control unit (CCU) responsible for updating the stimulation protocol based on sensed HR data and transmitting the updated commands to the handheld device. The implant is powered and controlled using the 13.56 MHz ISM band. It has a diameter of 15 mm and weighs 0.952 grams, including cuff electrodes. A custom-designed system-on-chip integrated within the implant delivers stimulation pulses as short as 5 $\upmu$s^44^. The handheld device, equipped with a rechargeable battery, can wirelessly control the implant and communicate with the CCU via Bluetooth. The CCU receives ECG data from the wearable sensing patch and adjusts the stimulation parameters based on the HR response. Using the FAW-VNS system, we explored chopped pulses with varying numbers of pulses and duty cycles to minimize bradycardia during VNS. The efficacy of the system was demonstrated by performing VNS and achieving the neural fulcrum in four anesthetized pigs.

## Methods

### Stimulator construction

The implant is made of a flexible polyimide board that houses the stimulator chip, WPT coil, surface mount devices (SMDs), and cuff electrodes. The chip is designed and fabricated using a standard 180-nm CMOS technology. The chip harvests power from the coil using a 5-stage half-wave rectifier. The coil is simulated in Ansys HFSS (HFSS 2021R1, Ansys Inc.^45^), and the inductance is simulated to be 2.93$~\upmu$H with a quality factor of 55. In addition, HFSS simulations for specific absorption rate (SAR) indicated that the SAR was well below the 10 W/kg limit specified by IEEE Std C95.1-2005. Supplementary Fig. S1 presents details of these simulations. The SMDs include a tuning capacitor for the coil, a storage capacitor for the chip, an optional diode as a visual stimulation indicator, a charge-balancing capacitor, and a discharge resistor. The cuff electrodes (Livanova) and the SMD components are assembled on the PCB using silver epoxy (EPO-TEK, H20E). The implant is finally packaged with biocompatible UV-cured epoxy (Master Bond UV15X-6Med-2).

### Realtime FAW-VNS system—sensing, CCU, and stimulation

The HR sensing patch consists of a Lead-I configuration patch ECG sensor. The patch electrodes are connected to an analog amplifier on an Arduino shield (Olimex Arduino Shield, Olimex Lmt). The shield is housed on an Arduino Due board, which digitizes the analog ECG at 200 Hz and interfaces to the CCU. To record the ECG data, the trigger signal synchronizes the HR sensing patch to the CCU. The Arduino buffers the analog data for 5 s and sends it to the CCU upon the trigger signal.

The real-time CCU is implemented in the MATLAB Simulink (MATLAB 2022b, MathWorks, MA^46^) environment with a state-based controller. The CCU maintains synchronization between the HR sensing patch and the VNS device through trigger signals used for communication. Digital signal processing (DSP) within the CCU extracts the HR information from the ECG signal. All signal conditioning and processing is done in real-time in Simulink using FIR filters. The ECG signal is bandpass filtered using a 24th-order high-pass finite impulse response (FIR) filter with a 4 Hz cutoff and a 48th-order low-pass FIR filter with a 45 Hz cutoff. An adaptive R-R peak detection is used to obtain the instantaneous HR within the 5-second buffer. For robust operation and immunity to natural HR variations, the real-time HR is computed as a 5-s median of the R-R peaks (1 value is assigned to each 5-s window). The baseline HR is computed as the average of the two most recent HR values obtained before each stimulation phase.

The FAW-VNS’s handheld device receives stimulation parameters and control signals via Bluetooth from the CCU. The CCU clock synchronizes the FAW-VNS timings and controls the implantable VNS IPG, controlling the stimulation timing. The stimulation protocol includes a 14-second active phase followed by a 66-second rest phase. Stimulation parameters span a frequency range of 1 Hz–20 Hz, PW from $64~\upmu$s-$1024~\upmu$s (at 100% duty cycle), and duty cycles ranging from 12.5% to 100% (12.5, 25, 50, 100), using two pulson stimulation waveforms. After each stimulation cycle, the CCU transmits the next set of stimulation parameters to the handheld device via Bluetooth for the subsequent stimulation.

### Surgical procedure

In-vivo studies were conducted on five pigs (Sus scrofa (Yorkshire), n = 5, 4 male & 1 female, adult, weighing between 49-55 kg, purchased from S&S Farms (currently Premier BioSource)) under terminal procedure to evaluate the performance of the FAW-VNS system. All animal studies were conducted under Animal Research: Reporting of In Vivo Experiments (ARRIVE) guidelines and were approved by the University of California, Los Angeles, Institutional Animal Care and Use Committee (IACUC). For the terminal studies, the animals were euthanized using sodium pentobarbital 100 mg/kg IV push following the approved IACUC protocol and the National Institutes of Health’s Guide for the Care and Use of Laboratory Animals. On the day of the terminal experiment, Tiletamine-Zolazepam (4–6 mg/kg, intramuscular) was used for the induction of anesthesia. Animals were endotracheally intubated and mechanically ventilated with a tidal volume of 400 to 600 mL and a respiratory rate of 12–16 breaths/minute. Anesthesia was then maintained with isoflurane vaporized (1–4% inhaled). A continuous rate infusion of fentanyl (0.2 $\upmu$g/kg/min) was started after induction and continued during the surgical procedures for analgesia. After induction of general anesthesia (vaporized isoflurane), the animal was positioned in dorsal recumbency. Indwelling catheters were percutaneously placed using ultrasound guidance in both femoral arteries, for blood pressure and blood gas monitoring, and femoral veins. The animal was instrumented with a gold standard ECG and a pulse oximeter. A spirometer was connected to the tracheal tube. The animal was mechanically ventilated using pressure control mode for the duration of the surgery. Body temperature was maintained using a hot air warming system if necessary. Lactate ringer fluid therapy at a rate of 5 ml/kg/h was administered intravenously throughout the procedure. Following the completion of surgical procedures, anesthesia was transitioned from isoflurane to alpha-chloralose (25–50 mg/kg/hour, intravenous), an anesthetic that does not depress cardiac and autonomic reflexes. Routine anesthesia monitoring included vital parameters such as ECG and invasive arterial blood pressure, central venous pressure, end-tidal CO2 (EtCO2), end-tidal isoflurane (Et Iso), pulse oximetry and core body temperature done through a rectal probe. The anesthetized animals were monitored to maintain physiological parameters within the normal limits. Anesthesia was adjusted accordingly by the anesthetist. The ventral neck region was clipped and aseptically prepared using chlorhexidine-based solutions. Longitudinal 20 cm skin incisions were made using monopolar electrocautery centered immediately to the left and right of the trachea. The incision was continued through the subcutaneous tissue and the sternohyoideus musculature until encountering the carotid sheath and vagus nerve. A 5–7 cm long segment of the left and right vagus nerves were circumferentially isolated by blunt dissection to allow placement of a VNS cuff. The vagal cuff was placed around the nerve by carefully sliding the cuff over the nerve, achieving good contact between them. The VNS cuff was placed at the mid-cervical level, approximately 2–3 cm from the nodose ganglion, while keeping the most consistent placement of the cuffs between animals. All animal experiments were performed in accordance with the relevant guidelines.

### Left ventricular pressure (LVP) data analysis

LVP data analysis was performed using MATLAB. The HR was obtained from the periodic time separation between the rising edge of the LVP waveform. Smoothing and fitting are performed to suppress the noise and track HR variation during the experiments.

### Electrochemical impedance spectroscopy (EIS) measurement and data analysis

EIS was measured using a PalmSense4 device. The PalmSense4 device was connected to the cuff electrodes wrapped taut around the vagus nerve. The reference electrode (RE) and counter electrode (CE) were shorted, and the working electrode (WE) and RE were connected to the cuff electrodes. The EIS is measured from 1 Hz to 10 kHz using a 10 mV AC voltage. A circuit fitting model was performed using PSTrace5.8 software. Levenberg–Marquardt algorithm was used with a lambda initial value of 0.01 and a scaling value of 10.

### Statistical analysis

The non-parametric Mann-Kendall test for trend was computed to determine the trend in HR response to the FAW-VNS system. The confidence level for the analysis was set to 95%. The Mann-Kendall test was performed per ref.^47^. Data analysis was performed in MATLAB using custom scripts.

### Figure illustration

**Figure 1:**
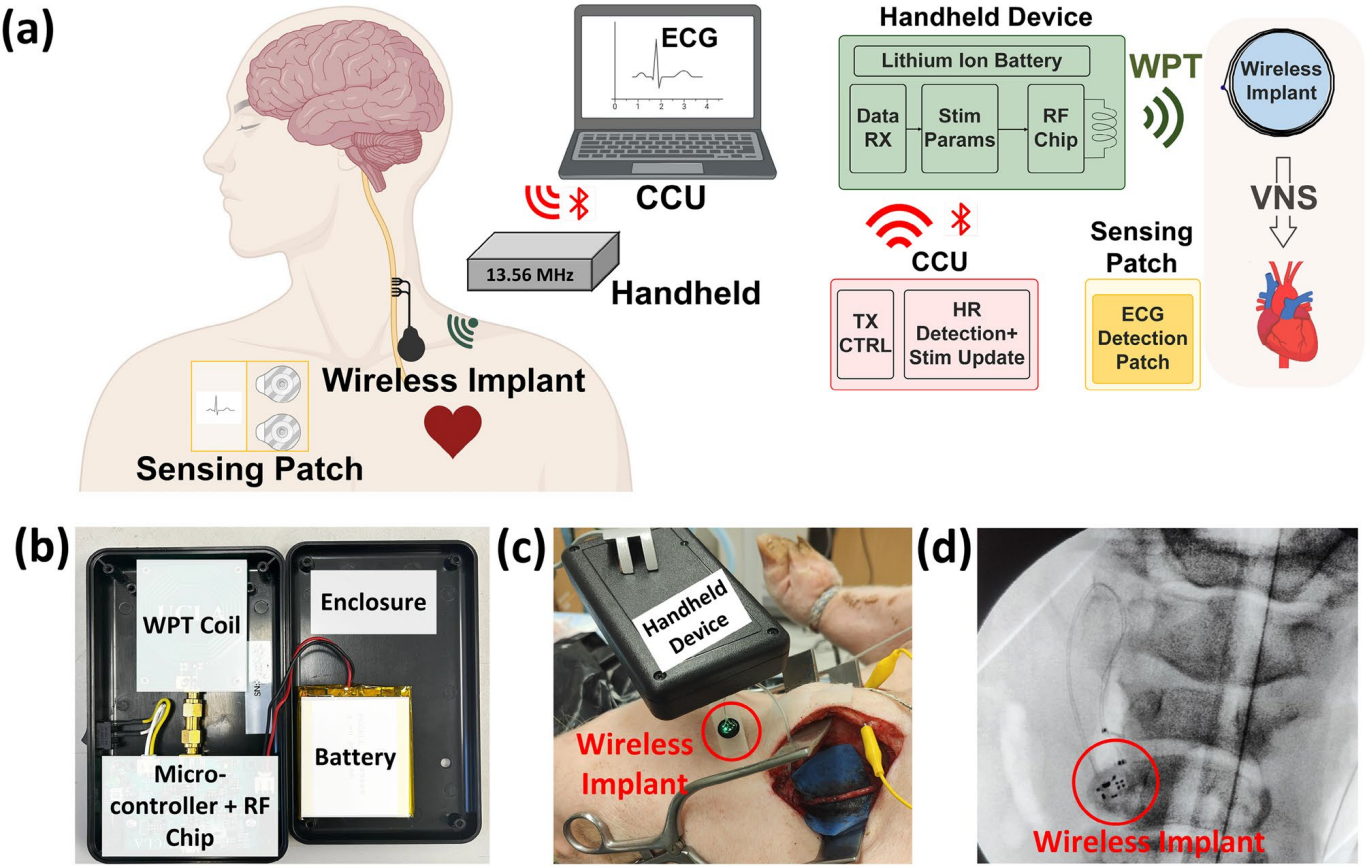
FAW-VNS system concept and implementation. (**a**) The conceptual diagram of the proposed FAW-VNS system and the block diagram of the closed-loop system (created with BioRender.com). (**b**) Internal view of the handheld device. (**c**) In-vivo validation of the implantable stimulator using the handheld device. (**d**) X-ray image of the implanted device.

Figure 1a was created with BioRender, BioRender.com. Data plotting is performed using MATLAB and OriginPro (OriginPro 2025, OriginLab Corporation, MA^48^). The figures are compiled in Visio (Visio Professional 2024, Microsoft Corporation^49^).

## Results

### FAW-VNS: system description

The conceptual picture of the proposed FAW-VNS system and its block diagram are shown in Fig. 1a. The handheld device transmits both power and stimulation patterns via resonant NRIC at the 13.56 MHz industrial, scientific, and medical (ISM) frequency band. This choice of frequency establishes a fair trade-off between coil size, WPT safety, and link bandwidth^43^. The stimulation pattern includes frequency, PW, and number of pulses, which are transmitted using a handheld device. The sensing patch records the ECG signal and transmits it to the CCU. The CCU processes the ECG signals and extracts the HR response. After each stimulation, the CCU updates the handheld device to converge to the neural fulcrum. The CCU communicates to the handheld device via Bluetooth and thereby closes the loop. The control algorithm is implemented in the MATLAB framework as part of the CCU. The handheld device is presented in Fig. 1b and consists of a WPT coil, an RF chip, a microcontroller, and a rechargeable battery. It has dimensions of 8 cm $\times$ 14 cm $\times$ 2.5 cm and weighs 170 grams. The coil is driven by the RF chip with modulated 13.56 MHz signals. A microcontroller is used for Bluetooth communication and modulating the RF chip. A 2500 mAh rechargeable battery enables portable operation, with a mini USB charging interface supporting extended use. The handheld device can operate continuously for up to six hours on a full charge. The sample setup and real-time operation of the FAW-VNS are presented in Supplementary videos S1 and S2. An in-vivo demonstration of the implant powered by the handheld device is shown in Fig. 1c. Figure 1d shows an X-ray image of the implanted chip in a sutured incision.

### Wireless implant

**Figure 2:**
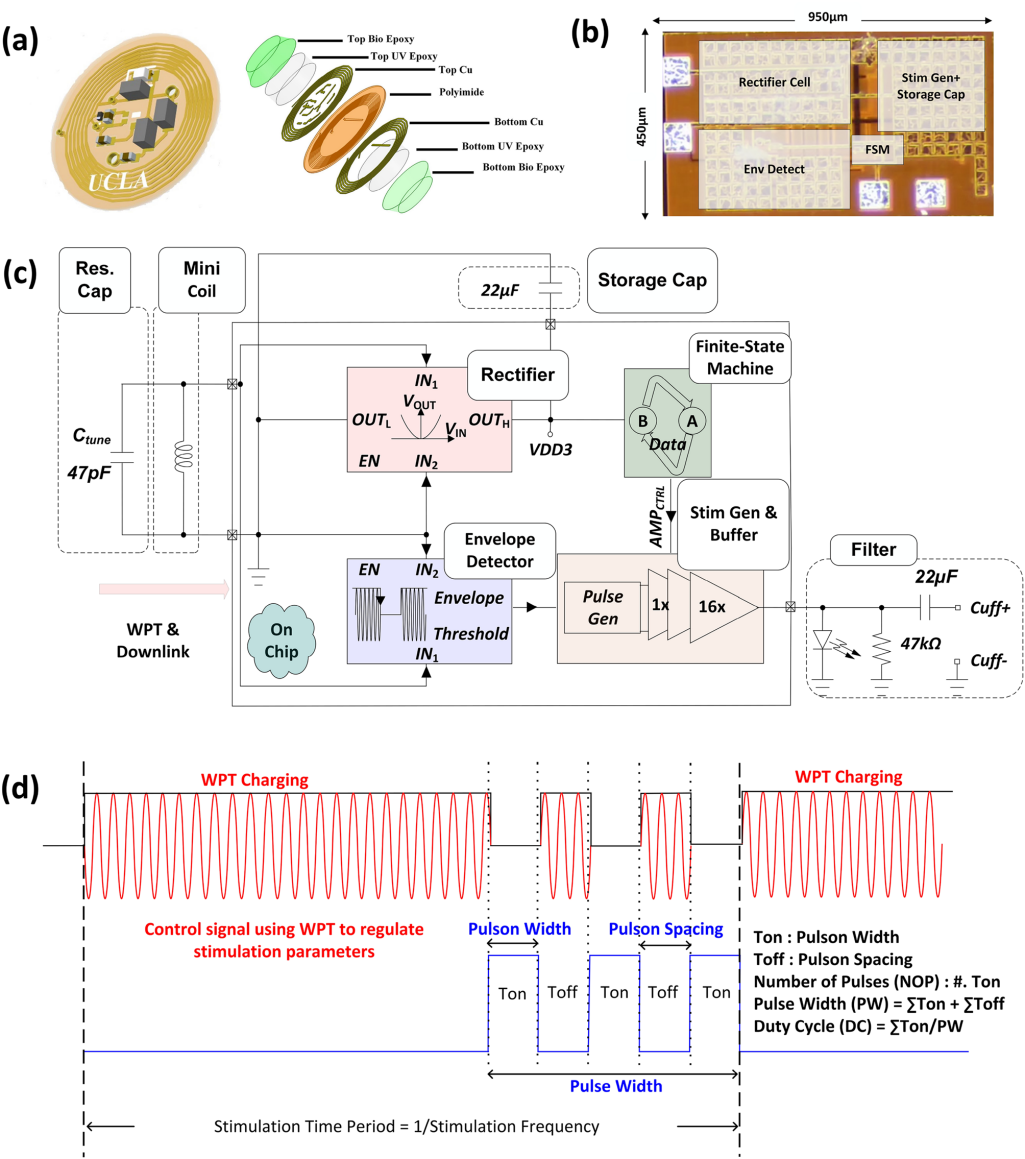
Implantable stimulator and its working principles. (**a**) Shows the 3D layer stack-up of the implantable stimulator. (**b**) The micrograph of the stimulator chip, including the WPT and finite state machine (FSM). (**c**) Schematic block diagram of the chip. (**d**) The control mechanism of the stimulator chip.

Conventional VNS systems broadly fall into two categories: current-controlled stimulation (CCS)^5,15^, or voltage-controlled stimulation (VCS)^43,50^. A comparison of the custom VCS stimulator with a conventional CCS stimulator in VNS applications is performed in ref.^50^. The CCS technique requires substantial circuit overhead to manage the cases with high impedances and limit the voltage inside the biological tissue^50^. Moreover, CCS complicates silicon integration as it necessitates special nodes to operate at large voltage levels. In contrast, VCS enables more straightforward silicon integration and supports low-power operation, but lacks precise control over the charge injected into the tissue because the stimulation current is dependent on the nerve’s impedance. The custom WPT VNS implant used in this study utilizes the VCS topology, which benefits from easy silicon integration and low-power, making it suitable for batteryless implantable operation. In the context of our closed-loop system, the need for precise charge control is mitigated, as stimulation parameters are continuously adjusted.

The chip is embedded in a circular implant with a diameter of 15 mm. The implant is made from a flexible and biocompatible polyimide substrate with a thickness of 0.18 mm. Figure 2a shows the exploded 3D view of the implant. The implant is tuned to the 13.56 MHz ISM band for WPT. Figure 2b,c shows the chip micrograph and circuit schematics of the chip, respectively. The on-chip rectifier harvests the RF energy and stores it on a 22-$\upmu F$ capacitor to provide a stable supply for the rest of the chip. The on-chip envelope detector extracts the envelope of the 13.56 MHz link using the nonlinear characteristic of the devices^51,52^. This envelope contains the stimulation pattern, as illustrated in Fig. 2d. The stimulation parameters are controlled via the envelope of the WPT signal. Specifically, turning the WPT signal “ON” halts stimulation, allowing appropriate control over stimulation characteristics such as the number of pulsons, PW, and pulson spacing. For a traditional rectangular waveform, the pulson spacing is zero, and the number of pulses is one^5^. The frequency of stimulation, which ranges from 1 Hz to 20 Hz, is defined by the repetition rate of each stimulation pattern. The chip produces a constant regulated pulson voltage between 2.6 V and 3.7 V via an on-chip voltage regulator. The regulator senses the harvested voltage and keeps it within operational range by stopping the stimulation when the voltage on the capacitor drops below 2.6 V or by dissipating the excess harvested power on a resistor when the harvested power is large. This regulated voltage level produces currents of about 3 mA (impedance measurements are provided in the Benchtop verification section), which is sufficient for therapeutic VNS^5^. The pulse duty cycle is defined as the ratio of the sum of individual pulson widths to total PW, as shown in Fig. 2d. By controlling the pulse duty cycle, we control the charge injected per stimulation. A lower duty cycle yields a lower charge injection, thus controlling the nerve activation. An on-chip non-overlapping pulse generator is designed to discharge the electrode after each stimulation. The non-overlapping logic ensures the discharge path is off during stimulation, preventing any short-circuit currents in the stimulator.

There has been a significant interest in improving the charge efficiency^53,54^ and fiber selectivity^55–57^ in VNS. In ref.^53,54^, authors explore prefiltering and pulse shaping to improve charge efficiency. However, these methods introduce significant circuit and power overhead, making them less suitable for wireless implantable systems. Fiber selectivity is crucial in targeting specific fiber types within the vagus nerve bundle^55–58^. In ref.^56^, the authors showed that for vagus nerve short-duration chopped pulses could preferentially stimulate C fibers compared to the A fibers (when compared to a continuous rectangular current pulse), enhancing the selectivity of VNS. In our work, we mainly explore VNS with two pulson chopped pulses. The effect of chopped pulses on HR in the subject (pig) is shown in Supplementary Fig. S2.

### FAW-VNS control algorithm

**Figure 3:**
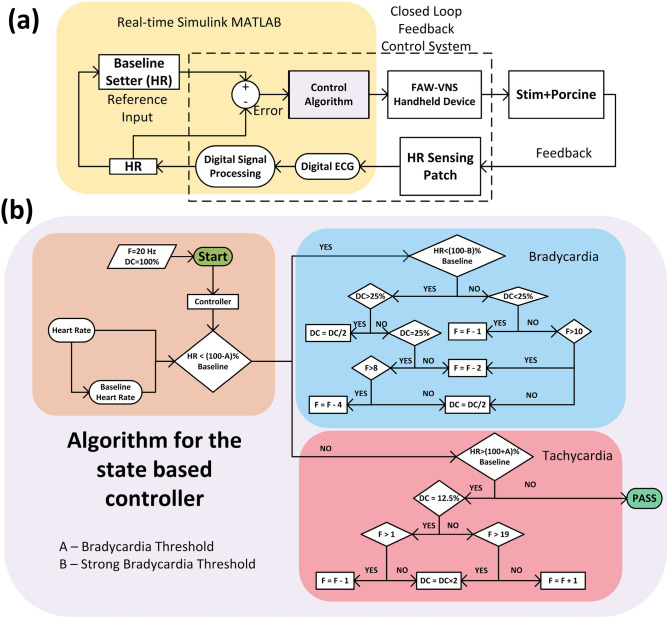
Control strategy and algorithm. (**a**) Depicts the overall control system, and (**b**) shows the logic of the state-based controller.

The bradycardic effect of VNS has been extensively studied and well documented in the literature^5,43^. At high stimulation intensities and frequencies, the bradycardia side effects can hurt the patient and limit its feasibility for routine daily use^59^. Consequently, there is an unmet need for unsupervised control of VNS that optimizes stimulation parameters to minimize off-target bradycardia. This objective can be accomplished through a closed-loop control mechanism that dynamically adjusts stimulation parameters based on the HR response. The block diagram of the closed-loop control approach is shown in Fig. 3a, which is implemented in MATLAB Simulink with the real-time toolbox. The closed-loop system (marked in a dashed envelope in Fig. 3a) is synchronized with the system clock of the CCU. The control algorithm takes HR inputs and a baseline HR to produce updated stimulation parameters for the handheld device. The stimulation protocol follows the standard VNS protocol as described in ref.^5^ with 14 s “ON” time (active phase) of stimulation followed by a 66 s “OFF” time (rest phase). The stimulation frequency and duty cycle vary from 1 Hz to 20 Hz and 12.5% to 100%, respectively.

In this work, we implement a state-based, discrete-time control algorithm, as illustrated in Fig. 3b, implemented in the MATLAB Simulink environment. The algorithm is designed to update a single stimulation parameter (either duty cycle or frequency) during each resting phase. By maintaining constant parameters throughout the active phase, the system avoids algorithmic instability. Incremental updates to the stimulation parameters enable gradual and accurate convergence to the neural fulcrum, while also allowing sufficient time for manual intervention in case of instability. The HR sensing patch records the ECG for a 5-second interval and buffers it to the CCU upon trigger. HR is then decoded via DSP in the CCU. For robust operation and immunity to natural HR variations, the real-time HR is computed as the median of R-R intervals within each 5-second buffer (yielding one HR value per window). The baseline HR is computed as the average of the last two HR values right before each stimulation phase. The prototype control algorithm is designed to achieve HR control within 2-4% of the baseline. The bradycardia threshold is set to one-fifth or one-tenth of the maximum HR response to VNS. This threshold exceeds the average natural HR variation calculated over a 5-s window during the rest phase. When the controller detects that the HR is below the threshold during VNS, it adjusts either the duty cycle or the frequency in the subsequent active phase. Likewise, in the case of tachycardia, the controller adjusts the duty cycle or frequency to counter it. The specific parameter to be updated and the magnitude of change are determined by the current state and the observed HR response during VNS.

### Benchtop verification

**Figure 4:**
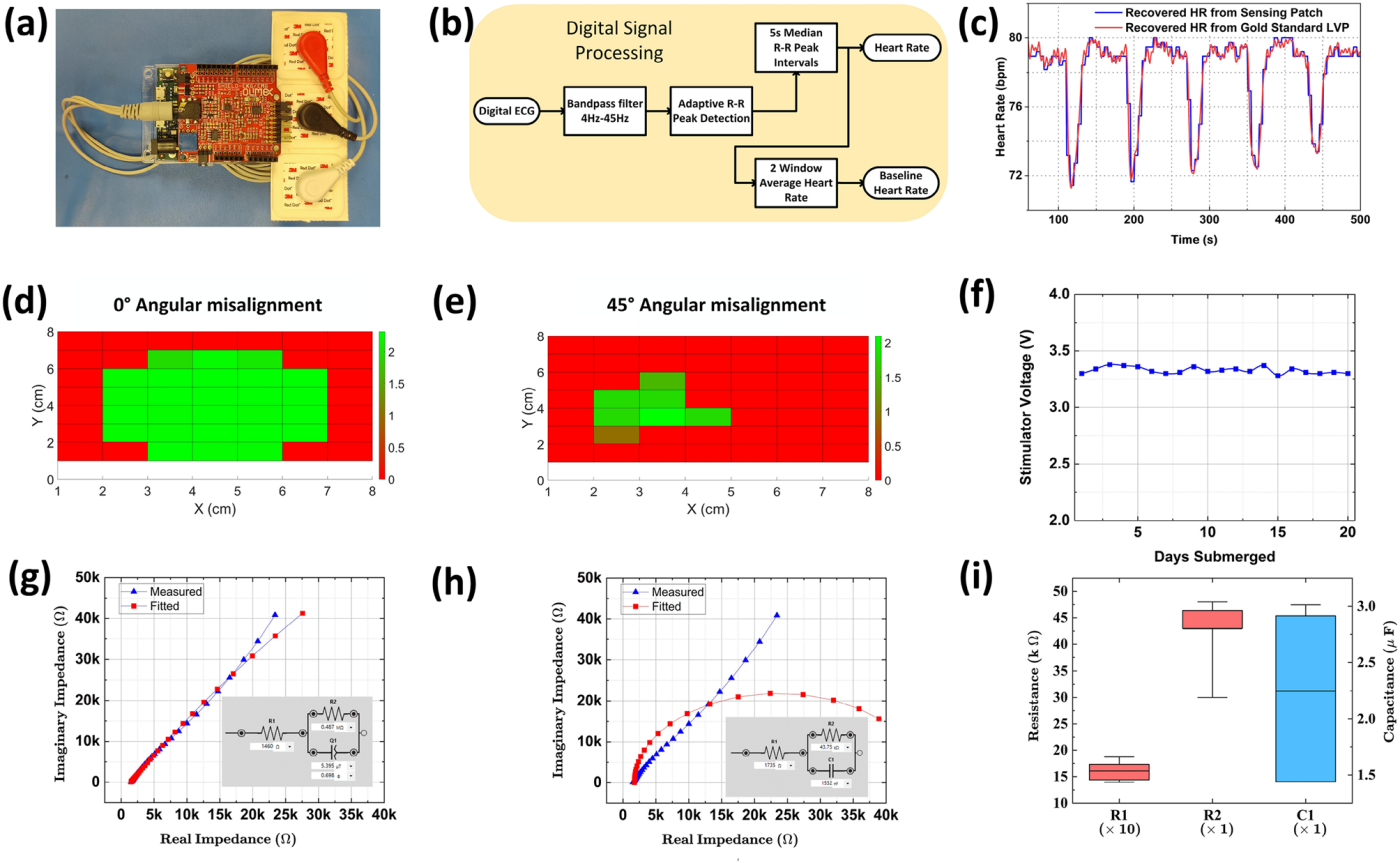
Benchtop verification. (**a**) The HR sensing patch. (**b**) The block diagram of the digital signal processing in Simulink to process HR. (**c**) Comparison of the HR extracted from the sensing patch and the gold standard medical LVP. (**d**) Effects of the lateral misalignment without and (**e**) with $45^\circ$ angular misalignment at 5 cm vertical distance between the WPT coil and stimulator. (**f**) Displays the stimulator working chronically after 20 days of submersion in PBS. Right vagus nerve EIS measurement and fit with (**g**) constant phase element model and (**h**) R-C fit models to characterize the impedance. (**i**) The ranges of component R-C fit values for EIS measurements in 4 different animals.

The ECG signal is sensed using surface patch electrodes and amplified using an Arduino shield (Olimex EKG-Shield, Olimex Ltd) connected to an Arduino Due board, as shown in Fig. 4a. The Due board interfaces the physical sensor to the Simulink environment. Within Simulink, HR is extracted through DSP of the ECG signal, which includes bandpass filtering and adaptive R-R peak detection, as shown in Fig. 4b. To validate the accuracy of the HR measurements from the sensing patch, the extracted HR is compared against a gold-standard measurement obtained from a medical-grade LVP sensor, as presented in Fig. 4c. Compared to gold standard equipment, we reach an average error (Eq. 1) of 0.50% after measuring HR for 700 s.

1 $$\begin{aligned} Average~HR~Error = mean\left( \frac{\vert HR_{Sensed} - HR_{LVP}\vert }{HR_{LVP}}\right) \times 100\% \end{aligned}$$

To verify robust operation for in-vivo experiments and future chronic studies, we perform measurements under misalignment, submersion, and implant loading conditions. In a realistic WPT scenario, the WPT coil and the implant will have an inevitable misalignment. Hence, to evaluate the performance of the wireless stimulator, a 2D scan for misalignment is performed with a vertical separation of 5 cm and external power of 2 W. Figure 4d shows the performance of the stimulator without angular misalignment and the ability to tolerate a lateral misalignment range of 5 cm in both X and Y dimensions. Introducing an additional angular misalignment of $45^\circ$, the operable range for lateral misalignment reduces to a 3-cm as shown in Fig. 4e. Furthermore, to verify the implant’s survivability in a biological medium, we submerged an implant in phosphate buffer solution (PBS) for 20 days and recorded the stimulation voltage. Figure 4f shows a consistent operation achieved after 20 days of immersion. We also verified the implants’ response to chopped pulses inside the PBS as shown in Supplementary Fig. S3. An EIS is performed to measure the impedance of the vagus nerve with the cuff electrode. This ensures the nerve loading is within acceptable levels for a 3.3 V stimulator in providing the necessary therapeutic levels of current^42^. Figure 4g,h show the Nyquist resistance and reactance plots of the right vagus nerves extracted using circuit fitting in PSTrace. Figure 4g displays the fitting with resistors and a constant phase element to fit the non-ideal capacitor behavior. Figure 4h shows the fitting with equivalent circuit models that are replicable in the laboratory (resistors and capacitors). The electrode resistance is only 330 $\Omega$, constituting a small portion of $R_{1}$ in the EIS measurements. The range of values after circuit fitting for four different animals is shown in Fig. 4i, verifying the loading conditions for our custom vagus nerve stimulator. The median value for for $R_{1}$, $R_{2}$, and $C_{1}$ are 1.58 k$\Omega$, 44 k$\Omega$, and 2.6 $\upmu$F with an average fitting error of 7.8$\%$, 16.6$\%$, and 9.4$\%$.

### In-vivo validation of open loop VNS

**Figure 5:**
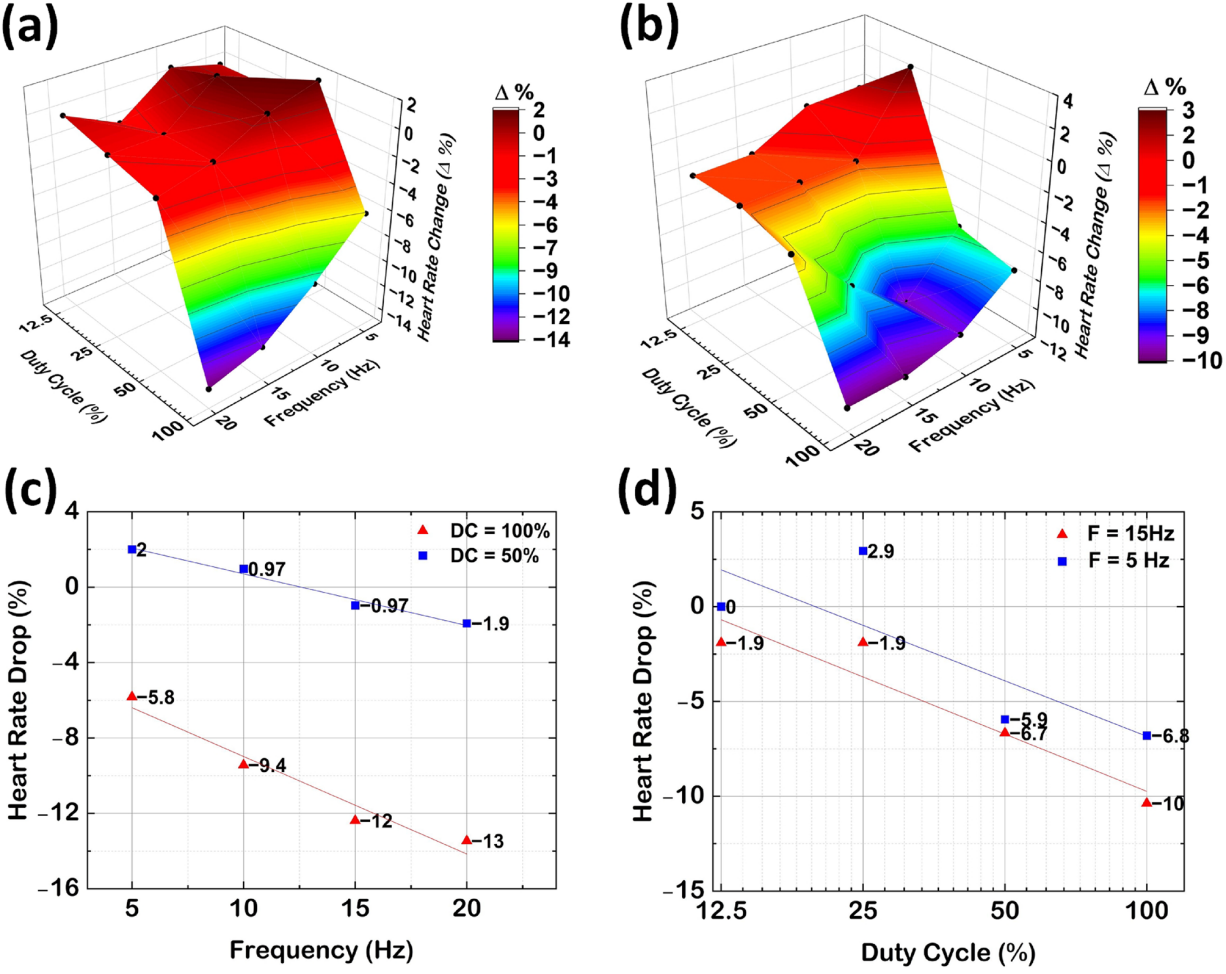
Open loop VNS. (**a**,**b**) shows the 3D surface plot of percentage change in HR upon stimulation for PW (at 100% duty cycle) of 256$~\upmu$s and 512$~\upmu$s. (**c**,**d**) shows the trends in HR changes with frequency for 256$~\upmu$s and duty cycle for 512 $\upmu$s.

In-vivo studies were conducted on pigs (Sus scrofa, female, adult, weighing 55 kg) under a terminal procedure to validate open loop VNS. The stimulation was applied to the right vagus nerve, and VNS was performed with frequency control ranging from 1 Hz to 20 Hz, a duty cycle control ranging from 100% to 12.5%, and PWs of either $256~\upmu$s or $512~\upmu$s. The stimulation protocol included a 14-s “ON” or active duration followed by a 66-s “OFF” or rest duration. The stimulators were wirelessly powered at a distance of 50 mm using the handheld device. Figure 5a,b illustrates the HR control using frequency and duty cycle as control parameters measured on the same animal (subject = 5) during a contiguous time window of a single measurement. For PWs of 256$~\upmu$s and 512$~\upmu$s, a consistent trend of bradycardia was observed at high frequencies and large duty cycles as shown in Fig. 5c,d. The severity of bradycardia decreased when the frequency or duty cycle was reduced. This is in agreement with previous studies reported in ref.^43^. The most significant bradycardia was observed at 20 Hz with a duty cycle of 100%, resulting in an approximately 13% reduction in HR. At lower frequencies and duty cycles, the HR change decreased to within 2% of baseline levels, which is considered a steady state for the controller during automated studies.

### In-vivo validation of closed-loop FAW-VNS

**Figure 6:**
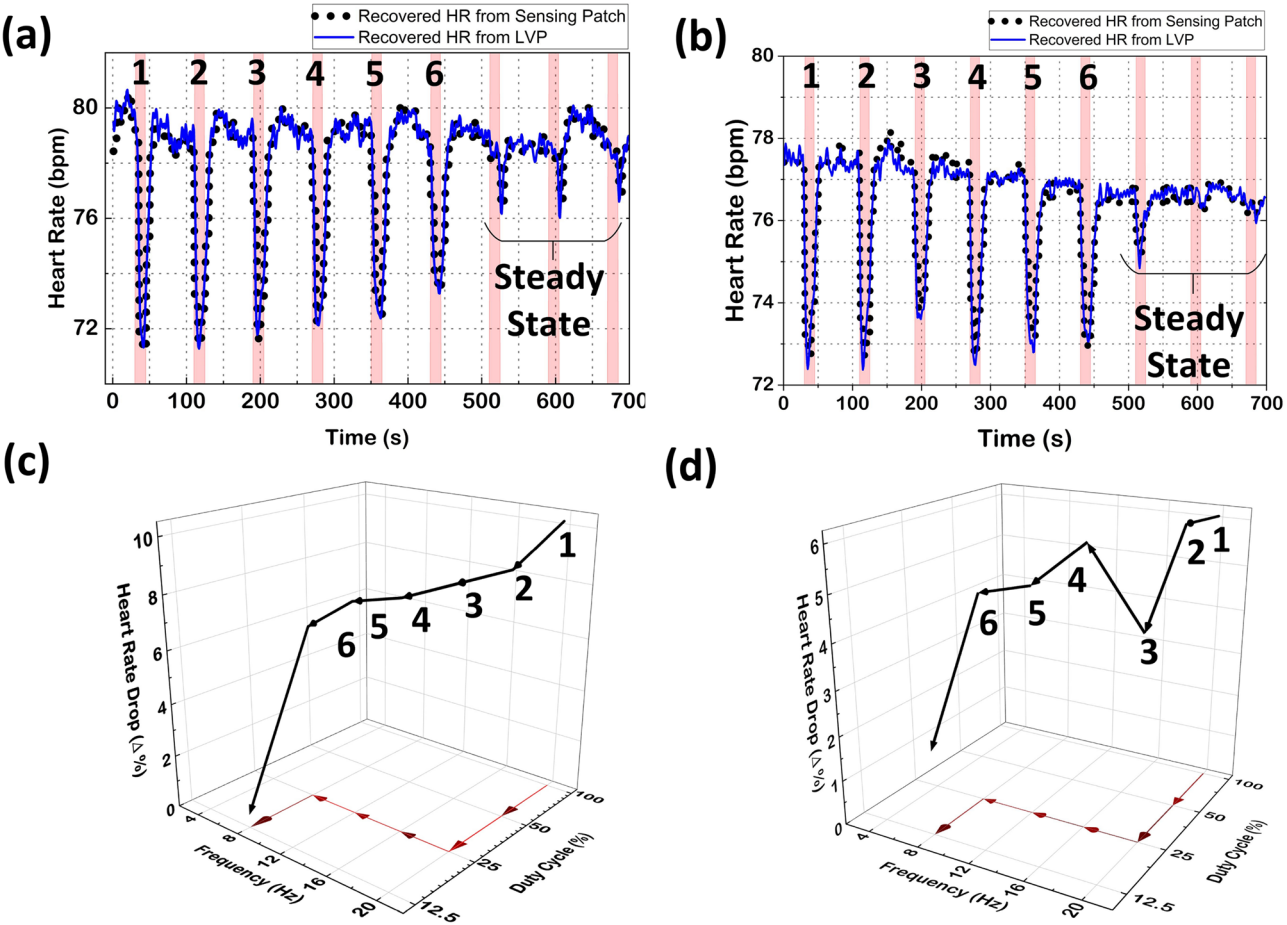
In-vivo validation of the FAW-VNS system. Transient HR response and achieving steady state neural fulcrum in (**a**) open incision and (**b**) closed incision. Flow of duty cycle and frequency of stimulation after each update in (**c**) open incision and (**d**) closed incision.

The goal of FAW-VNS is to demonstrate the closed-loop control after initializing it under adverse conditions with the highest stimulation frequency (20 Hz), PW ($1024~\upmu$s), and duty cycle (100%) to induce a significant bradycardic event. Following this initialization, the automated system is tasked with adjusting the stimulation parameters to maintain HR changes within a defined steady state range. Figure 6 displays HR responses in (a) an open-incision and (b) a closed-incision setting, recorded in the same subject (subject=2) during RVNS. The regions shaded in red indicate the active phase of stimulation. Figure 6c,d elaborate on the parameter adjustments made by the algorithm, showing systematic changes in duty cycle and frequency to reduce HR changes during VNS, demonstrating the algorithm’s effectiveness. The algorithm successfully achieved steady-state operation by adaptively tuning stimulation parameters in an automated closed-loop manner, without the need for human intervention. The initial HR reductions were 10% and 6% in the open and closed incision, respectively, and less than 2.5% after reaching the steady state. The residual HR decrease indicates the stimulation remained above the activation threshold of the nerve, confirming sufficient charge injection. Additional closed-loop studies performed on other Sus-scrofa (subjects 1,3, and 4) using the FAW-VNS system are provided in Supplementary Fig. S4.

## Discussion and conclusion

VNS has been explored for the treatment of several conditions, such as epilepsy, drug-resistant depression, cardiovascular diseases, chronic pain, and obesity^60^. Traditionally, VNS is delivered in an open-loop fashion with constant monitoring by a qualified surgeon to tune parameters based on heuristic approaches^5^. This supervision is essential to prevent side effects during VNS, such as bradycardia, which can be disruptive to the daily activities of an individual undergoing therapeutic VNS^61,62^. As a result, VNS is typically confined to specialized clinical environments, limiting its applicability in point-of-care settings. Hence, there is a need for an automated closed-loop control to achieve stimulation parameters with minimal side effects. Such a system would enable personalized VNS therapy, facilitate real-time health monitoring, and reduce overall costs. Therefore, we propose the FAW-VNS for dynamically adjusting stimulation parameters to achieve the neural fulcrum where the side effects of VNS are minimized.

In this study, we leverage the advantages of both the ST and FLC models by implementing a state-based controller that is easy to control, has a lightweight implementation, and is easily human-interpretable. A state-based decision tree is constructed based on prior empirical observation of the HR response to VNS^5^. For each state, one stimulation parameter (either duty cycle or frequency) is updated to help visualize the effect of the VNS on the subject. Slowing the system response time by a single parameter update per stimulation phase serves multiple purposes: (i) prevents large dynamic corrections, (ii) reduces the stress of rapid changes of stimulation parameters on the subject, (iii) improves stability of the control mechanism and (iv) promotes a smooth convergence to the steady-state operating point. The FAW-VNS system was validated in terminal studies using an adverse initial condition (bradycardia $\ge$10% induced by a 20 Hz frequency and 100% duty cycle) to display the attainment of the steady state. The state-based controller reached this steady state after 7 iterations in a fully automated manner. To statistically test the effect of the FAW-VNS system in finding the steady state, we perform the non-parametric Mann-Kendall test for trend. The trend is tested in the magnitude of the percentage drop in HR across 8 different FAW-VNS runs conducted in 4 different subjects. The Mann-Kendall tests the null hypothesis ($H_{0}$) of no trend against the alternative hypothesis ($H_{A}$) of a downward trend. The null hypothesis ($H_{0}$) is rejected in favor of the alternative hypothesis ($H_{A}$) if the Mann-Kendall statistic (S) is negative and the Mann-Kendall probability corresponding to the absolute value of S is less than a prior specified significance value ($\alpha$). For all the FAW-VNS runs, the S value was negative, indicating that the data later in time tended to be smaller. In 6 out of the 8 runs, the Mann-Kendall probability (p) was less than the significance value ($\alpha =0.05$), thereby rejecting the null hypothesis of no trend present. These results show that our proposed FAW-VNS system can reduce the HR drop significantly, with the average last stimulation percentage drop in HR 4 times lower than the first stimulation.

Additionally, to meet the strict challenges for a point-of-care therapeutic VNS, the stimulator used must be 1) untethered to maintain freedom of movement, 2) miniaturized to minimize discomfort and reduce surgical risk, and 3) wirelessly controlled to update the stimulation adaptively. Most present solutions use battery-powered implants, but these systems pose a plethora of drawbacks, such as the large form-factor, the need for frequent charging in clinical studies, and the risk of chemical leakage^23,25,27,63–65^, making them unfavorable from the point of care perspective. The proposed FAW-VNS system addresses the above-mentioned drawbacks and is preferred from the point of care perspective due to implant miniaturization, wireless operation, and the portability of the handheld device. This also enables untethered stimulation and freedom of movement.

Future studies would involve: (1) translating the algorithm capabilities to include chronic studies and account for the subject’s adaption or tolerance to VNS by performing titrations; (2) modifying the handheld device into a wearable necklace for improved point-of-care use; and (3) transitioning the CCU from MATLAB environment to a cell phone application, enabling data sharing with qualified medical personnel for longitudinal monitoring and therapy optimization.

## Supplementary Information

Supplementary Information 1.

Supplementary Information 2.

Supplementary Information 3.

## Data Availability

All relevant data are provided within the manuscript or supplementary information files. The datasets reported in the studies and subsequent analyses are available from the corresponding authors upon reasonable request.
