# Supplementary material for: A closed loop fully automated wireless vagus nerve stimulation system
Source: Sci Rep. 2025 Jul 30;15:27856. doi: 10.1038/s41598-025-11159-8 (PMC12311178; doi:10.1038/s41598-025-11159-8)
Supplement: Supplementary file 1 — Supplementary Information 1. [file 41598_2025_11159_MOESM1_ESM.docx]

Supplementary material

1. Supplementary Video S1
2. Supplementary Video S2
3. Supplementary Figure 1
4. Supplementary Figure 2
5. Supplementary Figure 3
6. Supplementary Figure 4





**Figure S1.** Supplementary: Wireless powered IPG package. HFSS simulation for (a) coil inductance, (b) coil quality factor (Q), and (c) HFSS specific absorption rate (SAR) simulation to assess WPT safety at 1 W power at 5 cm.


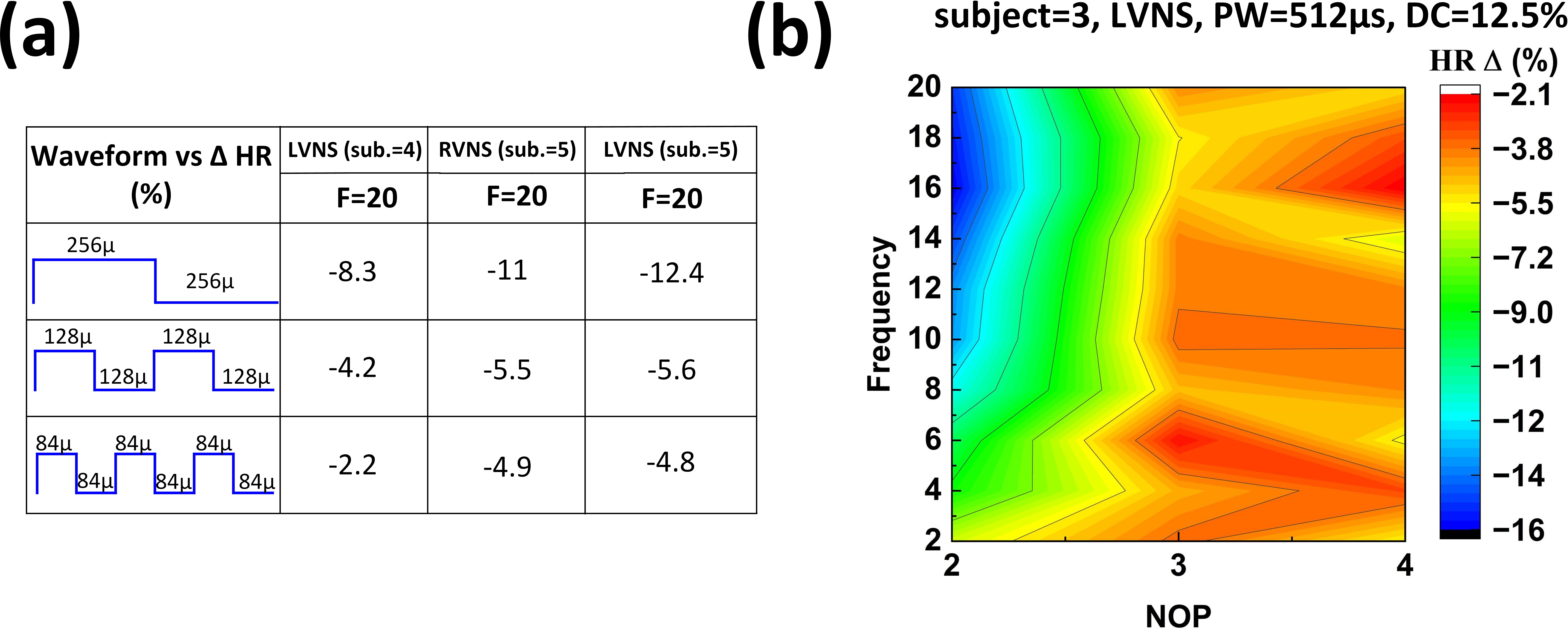


**Figure S2.** Supplementary: Effect of Chopped Pulses. (a) shows the percentage drop in HR on a single rectangular pulse and chopped pulses in a continuous window of a single measurement. (b) displays the effect of NOP on HR drop measured in a continuous window of a single measurement.


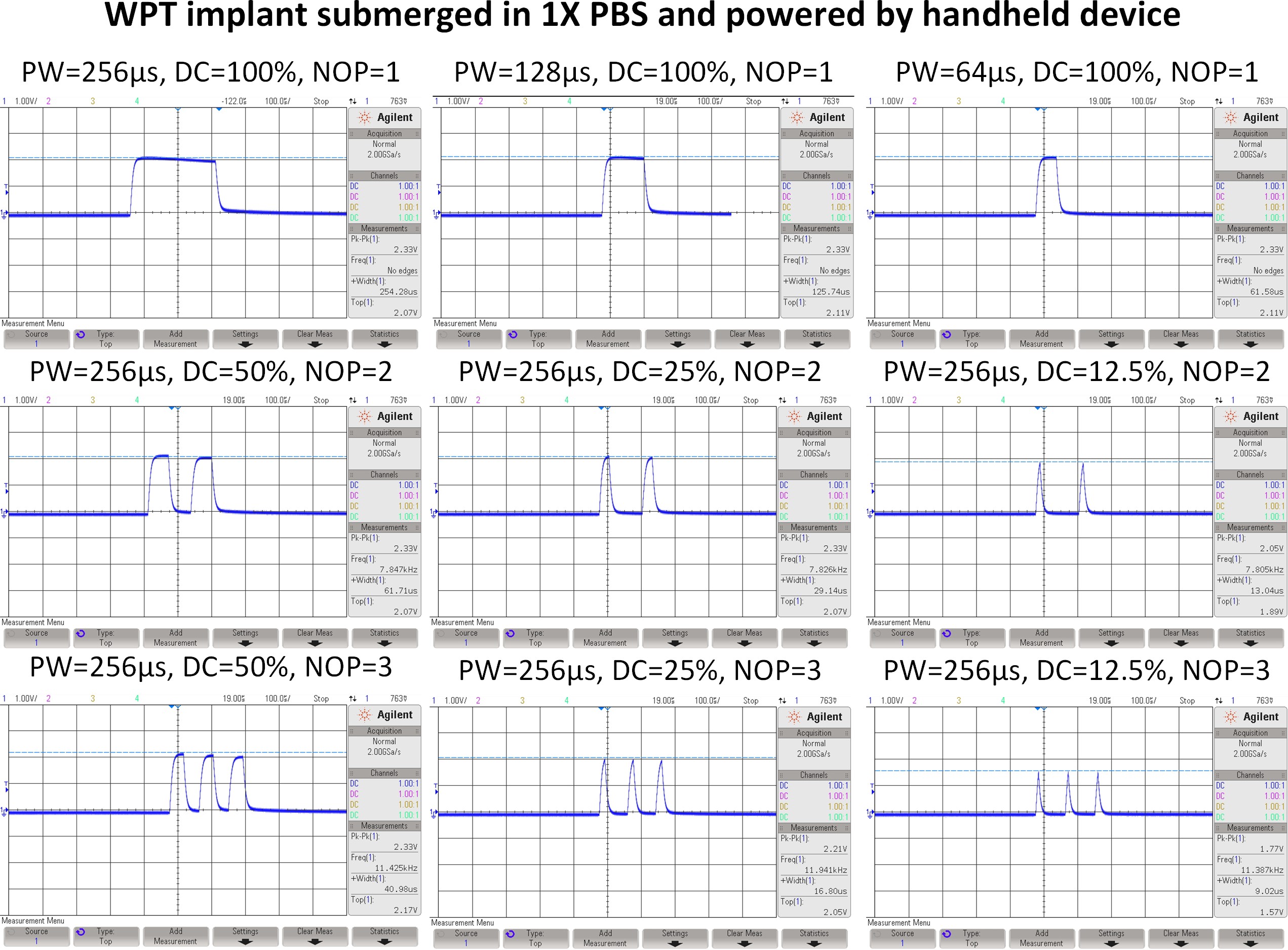


**Figure S3.** Supplementary: Benchtop test of the chopped pulses with WPT IPG submerged in PBS.





**Figure S4.** Supplementary: FAW-VNS validation in additional subjects. (a) and (b) show the FAW-VNS with frequency-only control (Duty Cycle = 100%), and (c) and (d) with both frequency and duty cycle control, and (e) and (f) with frequency and number of pulses (NOP).

**Supplementary Video Legends:**

Video S1: Automated closed loop VNS in closed incision 20 Hz 100% DC -> 16 Hz 25% DC with continuous monitoring.

Video S2: Sample setup of the wireless implant in PBS, with stimulation parameters of 20Hz, 50% DC, PW 256 µs.
